# Revisiting biomarker discovery by plasma proteomics

**DOI:** 10.15252/msb.20156297

**Published:** 2017-09-26

**Authors:** Philipp E Geyer, Lesca M Holdt, Daniel Teupser, Matthias Mann

**Affiliations:** ^1^ Department of Proteomics and Signal Transduction Max Planck Institute of Biochemistry Martinsried Germany; ^2^ Faculty of Health Sciences NNF Center for Protein Research University of Copenhagen Copenhagen Denmark; ^3^ Institute of Laboratory Medicine University Hospital LMU Munich Munich Germany

**Keywords:** biomarkers, diagnostic, mass spectrometry, plasma proteomics, systems medicine, Molecular Biology of Disease, Post-translational Modifications, Proteolysis & Proteomics, Systems Medicine

## Abstract

Clinical analysis of blood is the most widespread diagnostic procedure in medicine, and blood biomarkers are used to categorize patients and to support treatment decisions. However, existing biomarkers are far from comprehensive and often lack specificity and new ones are being developed at a very slow rate. As described in this review, mass spectrometry (MS)‐based proteomics has become a powerful technology in biological research and it is now poised to allow the characterization of the plasma proteome in great depth. Previous “triangular strategies” aimed at discovering single biomarker candidates in small cohorts, followed by classical immunoassays in much larger validation cohorts. We propose a “rectangular” plasma proteome profiling strategy, in which the proteome patterns of large cohorts are correlated with their phenotypes in health and disease. Translating such concepts into clinical practice will require restructuring several aspects of diagnostic decision‐making, and we discuss some first steps in this direction.

## Introduction

The central and integrating role of blood in human physiology implies that it should be a universal reflection of an individual's state or phenotype. Its cellular components are erythrocytes, thrombocytes, and lymphocytes. The liquid portion is called plasma, when all components are retained, and serum, when the coagulation cascade has been activated (blood clotting). For simplicity, we will use the term “plasma” rather than “serum”, since most conclusions apply to both.

**Figure 1:**
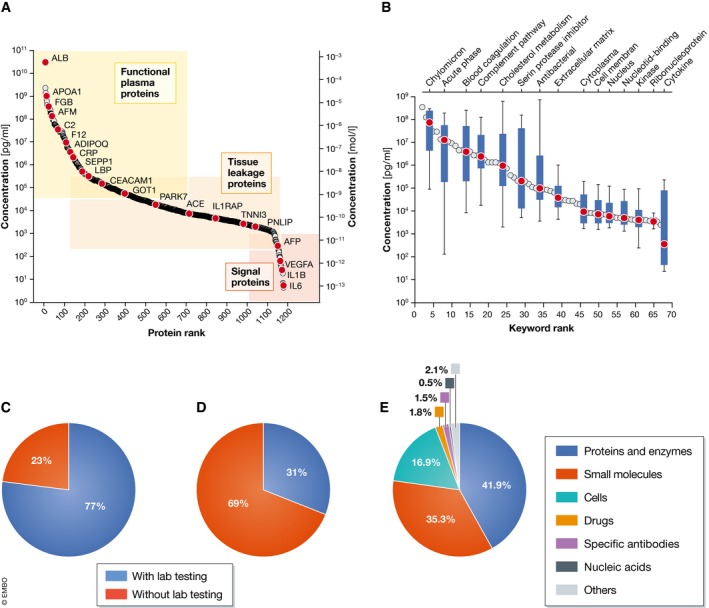
Blood‐based laboratory testing in a clinical setting (A) Concentration range of plasma proteins with the gene names of several illustrative blood proteins (red dots). Concentrations are in serum or plasma and measured with diverse methods as retrieved from the plasma proteome database in May 2017 (http://www.plasmaproteomedatabase.org/) (Nanjappa *et al*, 2014). (B) Bioinformatic keyword annotation of the plasma proteome database. The blue boxplots with the 10–90% whiskers visualize the range of diverse proteins contributing to distinct functions. (C) Percentage of inpatient admissions receiving blood‐based laboratory testing. Numbers are based on 9 million tests performed in the year 2016 at the Institute of Laboratory Medicine, University Hospital Munich. (D) Percentage of outpatient admissions receiving blood‐based laboratory testing. (E) Distribution of laboratory tests based on frequency of request. Examples of test for different classes of analytes are as follows: Proteins and enzymes*—liver enzymes, inflammatory proteins, tumor markers*; Small molecules—*electrolytes, substrates, vitamins*; Cells—*red, white blood cells, and platelets*; Drugs—*immunosuppressants, antibiotics, and drugs of abuse*; Specific antibodies—*autoantibodies and antibodies against infectious agents*; and Nucleic acids—*viruses and genetic variants*.

Concentrations of various plasma components are routinely determined in clinical practice. These include electrolytes, small molecules, drugs, and proteins. The proteins constituting the plasma proteome can be categorized into three different classes (Fig 1A and B). The first contains abundant proteins with a functional role in blood. These include human serum albumin (HSA, roughly half of total protein mass); apolipoproteins, which have crucial roles in lipid transport and homeostasis; acute phase proteins of the innate immune response; and proteins of the coagulation cascade. The second class are tissue leakage proteins without a dedicated function in the circulation. Examples are enzymes such as aspartate aminotransferase (ASAT) and alanine aminotransferase (ALAT), which are used for the diagnosis of liver diseases, as well as low‐level, tissue‐specific isoforms of proteins such as cardiac troponins. The third class are signaling molecules like small protein hormones (for instance, insulin) and cytokines, which typically have very low abundances at steady state and are upregulated when needed. Baseline levels of the cytokine interleukin‐6 (IL‐6) are 5 pg/ml, establishing a minimum 10^10^‐fold dynamic range of the plasma proteome when compared to the concentration of the most abundant protein, HSA, with about 50 mg/ml.

In accepted use, “a biomarker is a defined characteristic that is measured as an indicator of normal biological processes, pathogenic processes, or a response to an exposure or intervention” (FDA‐NIH: Biomarker‐Working‐Group, 2016). For the purpose of this review, we focus specifically on protein or protein modification‐based biomarkers. In this sense, there are more than 100 FDA‐cleared or FDA‐approved clinical plasma or serum tests, mainly in the abundant, functional class (50%), followed by tissue leakage markers (25%), and the rest include receptor ligands, immunoglobulins, and aberrant secretions (Anderson, 2010). Most of these are decades old, and the current introduction rate of novel markers is less than two per year (Anderson *et al*, 2013). A typical test consists of an enzymatic assay or immunoassay against a single target. Clinicians interpret the results in conjunction with other patient information, based on their expert knowledge. Ratios of abundances are only employed in specific cases. Examples are the 60‐year‐old De Ritis ratio of ASAT/ALAT to differentiate between causes of liver disease (De‐Ritis *et al*, 1957) or the more recent sFlt‐1/PlGF ratio for diagnosis of preeclampsia (Levine *et al*, 2004).

In contrast to enzymatic and antibody‐based methods, mass spectrometry (MS)‐based proteomics measures the highly accurate mass and fragmentation spectra of peptides derived from sequence‐specific digestion of proteins. Because the masses and sequences of these peptides are unique, proteomics is inherently specific, a constant problem with colorimetric enzyme tests and immunoassays (Wild, 2013). In principle, MS‐based proteomics can analyze all the proteins in a system—its proteome—and is in this sense unbiased and hypothesis‐free (Aebersold & Mann, 2016). Furthermore, MS methods are ideally suited to discover and quantify post‐translational modifications (PTMs) on proteins. These PTMs can also be the basis of diagnostic tests, such as HbA1c levels that serve as a readout of long‐term glucose exposure in the context of diabetes. Nevertheless, none of the routinely performed laboratory tests in plasma is based on proteins that were identified by mass‐spectrometric approaches, and in routine analysis, MS is so far only employed for measuring small molecules such as drugs and metabolites (Vogeser & Seger, 2016).

Over the past years, the technology of MS‐based proteomics has dramatically improved, and it is now a mainstay of all biological research that involves proteins (Cox & Mann, 2011; Altelaar & Heck, 2012; Richards *et al*, 2015; Zhang *et al*, 2016). In particular, its performance has robustly matured into a sensitivity and dynamic range that makes it interesting for biomarker studies. This review will focus on the prospects of determining proteins in blood by mass spectrometry. We start by empirically assessing the role of proteins in clinical diagnostic today and exhaustively review the literature on previous attempts at finding biomarkers in plasma by MS‐based proteomics. So far, proteomics strategies have involved extensive investigations of few samples, to be followed up by targeted approaches in larger cohorts. We discuss how recent advances in technology now enable a new strategy in which deep proteomes are measured for many time points and participants with the prospect to find new biomarkers and biomarker panels. We believe that proteomics will become part of the instrumental routine in the clinical laboratory within the next decade and may even eliminate current technologies in the far future.

## The current extent of clinical protein‐based diagnostics

Laboratory tests of blood and body fluids aim at disease diagnosis or confirmation, risk prediction, prognosis monitoring, and evaluating treatment effectiveness. It is commonly assumed that 70% of diagnoses are informed by blood testing, even though this number has not been well substantiated. At the Institute of Laboratory Medicine of the University Hospital Munich, laboratory testing is ordered for the vast majority of inpatients at some point during hospitalization (77%; Fig 1C). This fraction is much smaller in patients seen in one of the Hospital's outpatient clinics (31%; Fig 1D). These numbers indicate that hospitalized patients, who are usually sicker, are more likely to receive laboratory tests than ambulatory patients. Based on numbers of requested analyses, clinical routine is dominated by proteins (42% of analyses), followed by small molecules (35%) and cells (17%) (Fig 1E). Thus, already today proteins are the most frequently assayed class of laboratory analytes in clinical practice. We also note that methods suitable for determining plasma proteins have the largest share of the worldwide *in vitro* diagnostics.

Laboratory assays for plasma proteins are based either on classical clinical chemistry, utilizing enzymatic activities of certain plasma proteins, or on antibody‐based immunoassays. The costs of enzymatic assays are only in the cent‐range, and they run on high‐throughput automated analyzers, delivering up to 10,000 test results per hour. In contrast, immunoassays are more expensive (usually several euros/dollars per sample) and throughput of the respective automated analyzers is about 1,000 tests/hour. Large clinical chemistry as well as immunoassay‐based analyzers may carry reagents for more than 100 different analytical parameters. Main advantages of immunoassays are a greater degree of flexibility due to the accessibility to plasma proteins devoid of enzymatic activity and a significantly higher sensitivity. Another, clinically relevant issue is the time required per laboratory test. Due to the necessity of immediate decision‐making, the majority of enzymatic assays and several immunoassays have to be scaled down to analysis times of < 10 min. In general, immunoassays tend to take longer than enzymatic assays; nevertheless, the vast majority of current automated immunoassays require no more than 30 min.

## Systematic review of MS‐based plasma proteomics in biomarker research

Plasma proteins had already been investigated by two‐dimensional gel electrophoresis in the 1990s, sometimes in combination with MS identification of excised spots. However, these generally identified only a few dozen proteins, and as they preceded MS‐based proteomics, they are not discussed in this review. Claims of early cancer detection based on very low‐resolution MALDI spectra of plasma that produced patterns but no protein identifications (Petricoin *et al*, 2002) have not been substantiated (Baggerly *et al*, 2004), and these technologies have largely been abandoned today.

To obtain a comprehensive collection of publications dealing with plasma biomarker research and employing MS‐based proteomics, we performed an unrestricted PubMed search specifying co‐occurrence of the terms “biomarker”, “plasma OR serum”, “proteome”, “proteomics”, and “mass spectrometry”. This yielded an initial list of 947 publications of which 103 were reviews. We further subtracted studies that did not deal with human subjects or did not involve plasma or serum, leaving 381 original publications (Dataset EV1).

**Figure 2:**
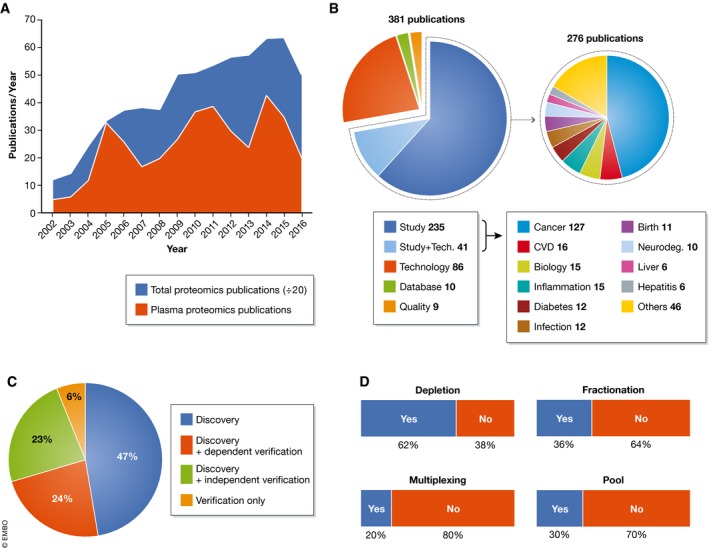
Comprehensive literature review (A) Publications using MS‐based proteomics in plasma biomarker research (red) compared to the total number of publications in proteomics (blue). (B) Pie charts about the intentions of the investigated studies and proportions of investigated diseases. (C) Overview of the percentage of studies, using discovery and validation phases. (D) Studies using pooled samples, depletion, fractionation, and multiplexing in plasma biomarker research using MS‐based proteomics.

Publications started to appear in 2002 and reached a maximum of 33 per year in 2005, when the special issue on the plasma proteome was released by the Human Proteome Organization (HUPO) (Omenn *et al*, 2005). Two further maxima appeared in 2011 and 2014 with 39 and 43 publications per year, followed by drops in 2013 to 24 and in 2016 to only 20 publications per year (Fig 2A). The observed dynamics contrasts with an ever‐expanding community of researchers using proteomics, which is reflected in thousands of publications per year, with a clear upward trend. The ratio of plasma proteome publications to total proteome publications is now < 1% and continues to drop. Given the clear medical need for plasma biomarkers and the success of MS‐based proteomics in other areas, this raises the question as to what holds back the field of plasma proteomics.

Of the 381 primary publications, about half dealt with the analytical descriptions of the workflow employed in plasma analysis, whereas the remainder investigated a physiological or pathophysiological question (Fig 2B). About a third of the latter focused on cancer, followed by cardiovascular disease (CVD), topics in human biology, inflammation, diabetes, and infectious diseases (Fig 2B). Clearly, this ordering reflects the interest in the diseases rather than the likelihood of finding relevant changes with the available technology. Only 47% of the studies had any kind of validation of the primary findings (Fig 2C). In half of the cases (24%), these were simple Western blots or ELISAs of candidate proteins performed with the same samples rather than an independent cohort as is usual practice in clinical studies. Only 36 papers used MS‐based proteomics to validate potential biomarkers that were proposed independently (Dataset EV1).

The extremely high dynamic range of plasma still makes it difficult to identify more than a few hundred of the most abundant proteins by LC‐MS/MS. To partially overcome this challenge, highly abundant plasma proteins are often depleted, generally through columns with immobilized antibodies directed against the top 1 to 20 proteins (Fig 2D). However, these antibodies are never entirely specific and bound proteins—such as HSA—themselves have an affinity for several other proteins (Tu *et al*, 2010; Bellei *et al*, 2011). Thus, the depleted plasma sample is not a quantitative representation of the original proteome. This is especially true when using “super‐depletion” (Qian *et al*, 2008)—a broad mixture of polyclonal antibodies raised against whole plasma—or beads with hexameric peptide mixtures that non‐specifically “normalize” the plasma proteome (Thulasiraman *et al*, 2005). Furthermore, these procedures introduce variability and additional expense into the workflow, generally precluding accurate quantification of plasma proteins. Therefore, their use is currently restricted to small discovery projects.

A second strategy to deal with the dynamic range and sensitivity challenge is extensive plasma fractionation, which can be done in various ways at the protein or peptide level. Several studies aiming at in‐depth coverage of the plasma proteome by combined depletion and extensive separation (up to hundreds of fractions) identified from several hundred to several thousand proteins (Liu *et al*, 2006; Pan *et al*, 2011; Cao *et al*, 2012; Cole *et al*, 2013; Keshishian *et al*, 2015; Lee *et al*, 2015). Note that many plasma proteome studies continue to use much less stringent statistical identification criteria than the 1% peptide and protein false discovery rates (FDR) that have become standard in MS‐based proteomics.

The decrease in throughput implicit in fractionation can partially be recovered by multiplexing. For example, between four and ten samples have been analyzed together using the iTRAQ or TMT strategies, in which samples are labeled with mass neutral tags that give rise to different low mass reporter ions (Kolla *et al*, 2010; Zhou *et al*, 2012; Cominetti *et al*, 2016). Quantification is achieved by fragmenting peptides and quantifying the relative ratios of the reporter ions (Bantscheff *et al*, 2008). Although attractive in principle, these techniques generally suffer from ratio distortion caused by co‐isolated peptide species that all contribute to the same reporter ion pattern (“ratio compression”). Regulation of very low‐level proteins or those with small but disease‐relevant changes may be completely obscured. In shotgun proteomics, eluting peptides are fragmented in order of intensity (data‐dependent acquisition), a semi‐stochastic process that may lead to missing values across LC‐MS/MS runs. Recently introduced data‐independent acquisition strategies more consistently identify peptides across runs (Picotti & Aebersold, 2012; Sajic *et al*, 2015). However, they are incompatible with reporter‐ion‐based multiplexing because one would quantify the average of groups of peptides.

In about 30% of the studies, plasma samples were pooled to reach a desired plasma proteome coverage within the available measuring time. This approach sacrifices within‐group variances and outlier or contaminant proteins in individual samples can skew the whole group, making it all but impossible to assess whether proteins that are different between groups are actually significant on a person‐by‐person basis.

Partly as a consequence of the demands on instrument time, generally no more than 20–30 samples were analyzed and only few exceeded 500 (Garcia‐Bailo *et al*, 2012; Cominetti *et al*, 2016; Lee *et al*, 2017). Considering the large number of measurement points within samples, these are small sample numbers. Accordingly, most studies proposed a few “potential biomarkers”, defined as proteins that differ between cases and controls. Furthermore, many of these candidates are unlikely to be specific indicators of the disease in question, because they belong to biological categories that are at best indirectly related to the disease or are likely artifacts of sample preparation (such as keratins and red blood cell proteins). In summary, limitations in proteomics technology and experimental design have prevented the identification of true biomarkers in the published literature to date. To our knowledge, the only possible exception is the OVA1 test, in which the levels of the highly abundant plasma proteins beta‐2 macroglobulin, apolipoprotein 1, serum transferrin, and pre‐albumin were combined with the previously established ovarian cancer marker CA125 in a narrow, FDA‐approved indication (Rai *et al*, 2002; Zhang *et al*, 2004).

## Triangular MS‐based biomarker discovery and validation strategy

**Figure 3:**
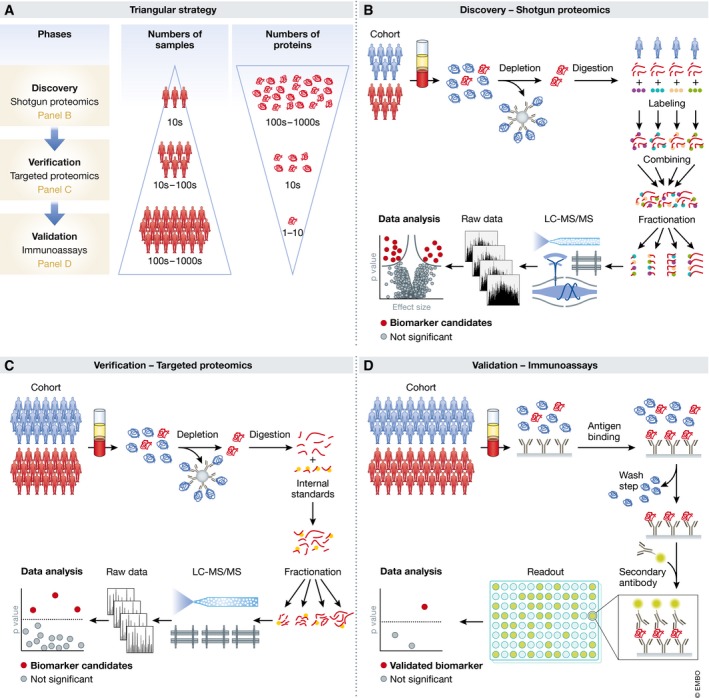
Current paradigms in plasma biomarker research (“triangular approach”) (A) A relatively small number of cases and controls are analyzed by hypothesis‐free discovery proteomics in great depth, ideally leading to the quantification of thousands of proteins (top layer in the panel). This may yield tens of candidates with differential expression that are screened by targeted proteomics methods in cohorts of moderate size (middle layer). Finally, for one or a few of the remaining candidates, immunoassays are developed, which are then validates in large cohorts and applied in the clinic (bottom layer). (B) Workflow for hypothesis‐free discovery proteomics. (C) Targeted proteomics for candidate verification. (D) Development of immunoassays for clinical validation and application.

The principal advantage of hypothesis‐free MS‐based proteomics is that no assumptions need to be made regarding the possible nature and number of potential biomarkers, in stark contrast to single protein measurements in classical biomarker research. Conceptually, MS‐based proteomics combines all possible hypothesis‐driven biomarker studies for each disease into one and furthermore defines the relation of potential biomarkers to each other. In practice, the challenges of plasma proteomics have so far prevented in‐depth and quantitative studies on large cohorts. Instead, a stepwise or “triangular” strategy for biomarker discovery has been advocated, with several phases in which the number of individuals increases from a few to many, whereas the number of proteins decreases from hundreds or thousands to just a few (Rifai *et al*, 2006; Fig 3A).

The typical workflow for hypothesis‐free discovery proteomics in plasma is similar to that used in other areas of bottom‐up proteomics (Aebersold & Mann, 2016; Altelaar & Heck, 2012; Fig 3B). Briefly, proteins are enzymatically digested into peptides, which are separated by high‐pressure liquid chromatography (HPLC) coupled to electrospray ionization. Peptide masses and abundances are measured in the mass spectrometer in full MS scans, whereas a further step of peptide fragmentation produces MS/MS spectra for peptide identification. Well‐established proteomics software platforms automatically and statistically rigorously identify peptides in database searches and quantify them (Cox & Mann, 2008; MacLean *et al*, 2010; Rost *et al*, 2014). Furthermore, plasma contains blood components such as lipids that can easily clog HPLC columns, which necessitates dedicated peptide cleanup procedures (Geyer *et al*, 2016a).

Targeted proteomics for candidate verification is a second phase of the triangular strategy (Fig 3C). A relatively small number of proteins (typically < 10) with differential expression in the discovery phase are tested in a larger and ideally independent cohort. Since immunoassays are often not available, targeted MS methods can be employed. The most widespread of these is “multiple reaction monitoring” (MRM—sometimes also called single or selected reaction monitoring—SRM) (Picotti & Aebersold, 2012; Carr *et al*, 2014; Ebhardt *et al*, 2015). For each protein, a set of suitable peptides is selected and their elution and fragmentation behavior is assessed to define an MRM assay. During analysis, the mass spectrometer is programmed to continuously fragment only these peptides as they elute. By monitoring several fragments per peptide, sensitive and specific quantification can be achieved even with low‐resolution mass spectrometers. The advantage of MRM over shotgun proteomics for verification is its higher sensitivity and throughput. Inter‐laboratory studies have achieved good reproducibility (Addona *et al*, 2009; Abbatiello *et al*, 2015), but reported sensitivities typically do not reach the low ng/ml concentration range and practically achieved multiplexing capabilities are limited to dozens of peptides (Percy *et al*, 2013; Shi *et al*, 2013; Oberbach *et al*, 2014; Wu *et al*, 2015). Nevertheless, two recent studies have reported the targeting of 82 and 192 proteins, respectively (Ozcan *et al*, 2017; Percy *et al*, 2017). The sensitivity of MRM can be improved to the low ng/ml or even high pg/ml ranges by more extensive sample preprocessing with depletion or fractionation (Burgess *et al*, 2014; Kim *et al*, 2015; Nie *et al*, 2017).

Absolute and accurate quantification requires internal standards—generally heavy isotope versions of the monitored peptides. Synthesized heavy peptides are added after digestion, creating a source of quantitative inaccuracy since the variability of protein digestion is not taken into account. This can be addressed by embedding the peptide in its original sequence context, for instance, in the SILAC‐PrEST strategy, in which a 150‐ to 250‐amino acid stretch of each protein of interest, fused to a quantification tag, is recombinant expressed in a heavy form (Zeiler *et al*, 2012; Edfors *et al*, 2014; Geyer *et al*, 2016a).

Targeted methods can also be combined with immuno‐enrichment of proteins or peptides. For instance, in “stable isotope standards and capture by anti‐peptide antibodies” (SISCAPA) specific peptides are immunoprecipitated together with their heavy‐labeled counterparts, followed by rapid MS‐based readout (Anderson *et al*, 2004; Razavi *et al*, 2016). This combines the enrichment capabilities of antibodies with the specificity of MS detection; however, development of assays can be difficult and time‐consuming—narrowing the advantage compared to purely antibody‐based methods.

The final phase in the triangular strategy is the validation with immunoassays, a field that has matured over decades. For maximum specificity, sandwich assays are typically preferred (Fig 3D). While they are costly and laborious to develop, they can achieve high sensitivity and high throughput. Even cohorts with thousands of participants can be tested with this technology, but only for one or a few candidate biomarkers. Such large numbers may be necessary to establish specificity not only against controls but also with respect to other diseases. Standard requirements include insuring adequate statistical power and replication in an independent population. Today, such clinical studies can be expensive multi‐year endeavors, partly explaining the paucity of new biomarkers.

Immunoassays have some inherent limitations, mostly related to antigen‐antibody recognition. These include cross‐reactivity, interference by background molecules such as triglycerides, and non‐linear response (“hook effect”) (Hoofnagle & Wener, 2009; Wild, 2013). Furthermore, not all clinically important protein variants are easily recognizable by antibody‐based assays. Given these limitations, MS‐based methods would be attractive alternatives in at least some large‐scale clinical trials, but this requires much more robust, sensitive, and higher throughput technologies than those available today.

Over the last decade, the proteomics community has developed guidelines for proper development of biomarkers that discuss quality standards and emphasize the importance of selecting adequate cohorts that ensure statistical significance of the findings as well as specificity of potential biomarkers and their potential clinical application (Luque‐Garcia & Neubert, 2007; Paulovich *et al*, 2008; Mischak *et al*, 2010; Surinova *et al*, 2011; Skates *et al*, 2013; Parker & Borchers, 2014; Hoofnagle *et al*, 2016).

Not surprisingly in view of the rigorous requirements of the triangular strategy, there are few, if any, reports in which it has been applied completely and successfully. This may also partly be due to the fact that three different technologies—shotgun proteomics, targeted proteomics, and immunoassay development—are involved. Many publications just describe the first phase or only combine it with immunoassay verification in the same cohort (Dataset EV1).

Among the studies with more than a few participants and with some verification, the majority selected candidates of interest and performed Western blotting, ELISA, or MRM assays. A representative example is the study by Zhang *et al* (2012) in which depleted plasma of 10 colorectal cancer patients versus controls was labeled with iTRAQ and fractionated, leading to the identification of 72 proteins. Among several up‐ or downregulated proteins, ORM2 was followed up by ELISAs in 419 individuals. Since this protein is a part of the innate immune system (like the other two upregulated candidates), it is unlikely to be a specific cancer marker. In another study, super‐depletion, iTRAQ labeling, and fractionation identified 830 proteins in a discovery cohort of 751 patients with cardiovascular events and controls that had been reduced to 50 pooled samples (Juhasz *et al*, 2011). The known markers CRP and fibronectin were selected from the list of candidates and found to be significantly upregulated in the original cohort by immunoassays against these proteins. In a heart transplantation study, analysis of depleted and iTRAQ‐labeled plasma from 26 patients at five time points before and after surgery identified a total of more than 900 proteins (273 per individual; Cohen *et al*, 2013). MRM assays and ELISAs against five medium‐abundant proteins in a partially independent follow‐up cohort of 43 individuals served to develop a computational pipeline for risk markers for organ rejection. In an approach of potential clinical utility, depleted plasma from a mouse model of breast cancer allowed the identification of more than 1,000 plasma proteins from which 88 were selected for MRM assays in an independent verification cohort of 80 animals (Whiteaker *et al*, 2011).

## Rectangular biomarker strategy and plasma proteome profiling

In the last few years, the community has substantially improved all aspects of the workflow of MS‐based proteomics. In sample preparation, laborious, multi‐stage preparation workflows have been replaced by robust, single‐vial processing with a minimum of manipulation steps. This also helps with automation and increases throughput. The sensitivity and sequencing speed of MS instruments have improved severalfold. The entire LC‐MS/MS system has become much more robust, although this is still far from what will be needed for routine clinical application. Finally, bioinformatic analysis of the results is now statistically sound and straightforward to use and increasingly enables correlation of MS results with a wide range of other classical clinical and additional “omics” data. Illustrating the power of cutting edge MS‐based proteomics, cell lines can now routinely be quantified to a depth of more than 10,000 different proteins in a relatively short time, sometimes even without any fractionation (Mann *et al*, 2013; Richards *et al*, 2015; Sharma *et al*, 2015; Bekker‐Jensen *et al*, 2017).

**Figure 4:**
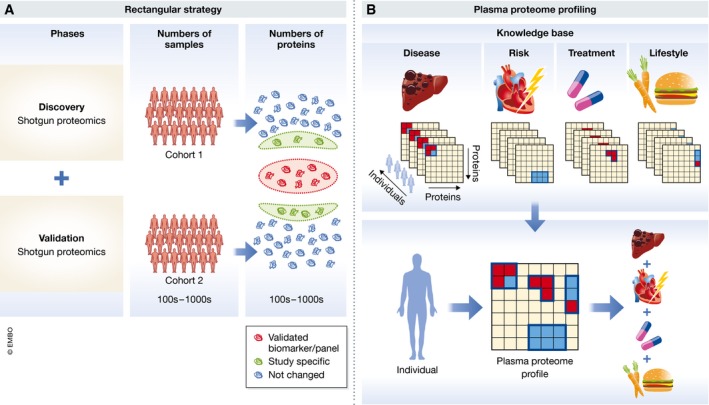
Rectangular workflow (A) A large cohort is investigated in the discovery phase with as much proteome coverage as possible. In the validation phase, another cohort is analyzed to confirm the biomarker candidates, but it uses the same technology and similar cohort size. Both cohorts can be analyzed in parallel, but only the proteins that are statistically significantly different in both studies (orange as opposed to green circle in the right‐hand part of panel A) are validated biomarkers. (B) Plasma proteome profiling of diverse lifestyle, disease, treatment, or other relevant alterations will over time build up a knowledge base that connects plasma protein changes to perturbations in a general manner (upper panel). The plasma proteome profile of a given individual can then be deconvoluted using the information and algorithms associated with the knowledge base (lower panel).

Given this technological progress of proteomics in cell line and tissue samples, we asked whether one could also develop a fast and automated workflow that would quantify the plasma proteome in depth in a large number of samples (Geyer *et al*, 2016a). We reasoned that this would then enable a “rectangular strategy” in which as many proteins as possible are measured for as many individuals and conditions as possible. In contrast to the triangular workflow, the initial discovery cohort would be much larger, ideally encompassing hundreds or thousands of participants, resulting in a greater likelihood to reveal any patterns that might differentiate the investigated groups or conditions. These larger initial numbers of plasma proteomes would allow the discovery of statistically significant, but small differences and changes associated with a group of proteins. In the proposed rectangular strategy, discovery and validation cohorts would both be measured by shotgun proteomics in great depth. This removes the dependency of validation on discovery, meaning that both cohorts can be analyzed together (Fig 4A). Moreover, having separate cohorts allows unmasking study‐specific confounders. A further advantage of the rectangular strategy is its ability to discover and validate protein patterns that are characteristic of particular health or disease states, in addition to single biomarker candidates, something that is unattainable with the triangular approach.

Interestingly, an analogous change of concept has already happened a number of years ago for genome‐wide association studies (GWAS). Researchers in this field found that joint analysis of as many samples as possible was superior to a sequential pipeline (Skol *et al*, 2006). In proteomics, the obvious challenge is achieving sufficient proteomics depth in a short time, ideally without depletion and in a robust workflow. This goal has not been achieved at the time of writing, but the current rate of technological improvements promises to make it feasible in the near future. Below, we discuss four examples of this emerging approach.

The first of these investigated a cohort of 36 monozygotic and 22 dizygotic twin pairs to determine the influence of genetic background on the levels of plasma proteins (Liu *et al*, 2015). The authors established a spectral library using depleted, fractionated, and pooled samples and measured their samples with data‐independent acquisition (DIA). A total of 232 plasma samples were then measured with 35‐min gradients in a data‐independent mode, leading to the consistent quantification of 1,904 peptides and 342 proteins. Interestingly, protein levels were often relatively stable within individuals as compared to between individuals. Furthermore, there were clear indications for the levels of some proteins to be under genetic control. For instance, processes connected to “immune response” and “blood coagulation” tended to be heritable, whereas those associated with “hormone response” did not. Although a pioneering study, the number of plasma proteomes analyzed was relatively small in view of the generality of the research question posed. Generally, genetics studies routinely investigate thousands of participants to tease out subtle heritable effects, illustrating the need for much higher throughput in clinical proteomics.

Malmström *et al* (2016) induced sepsis in mice by injecting *S. pyogenes* and followed their plasma proteomes through three time points on non‐depleted, non‐fractionated samples. A library of diverse mouse tissues was employed to support data‐independent identifications as well as to determine the origin of tissue damage proteins. In this way, 2‐h runs quantified an average of 786 mouse proteins, although it should be noted that proper FDR criteria for inferring peptide identities in the complex DIA MS/MS spectra are still being discussed (Nesvizhskii *et al*, 2007; Bruderer *et al*, 2017; Rosenberger *et al*, 2017). Several expected categories of plasma proteins increased during sepsis, as well as some markers associated with damage to the vascular system. Some of the changes were related to mobilization of the immune system against the pathogen, and others appeared to be correlated with necrosis in severely affected animals.

In a workflow termed “plasma proteome profiling”, we focused on the rapid and robust analysis of only 1 μl of undepleted plasma from a single fingerpick (Geyer *et al*, 2016a). Total gradient time was only 20 min, enabling extensive investigation of analytical, intra‐assay, intra‐individual, and inter‐individual variation of the plasma proteome. Based on the quantification of 300 plasma proteins, about 50 FDA‐approved biomarkers were covered with label‐free quantification (CV < 20%). Rapid analysis of a wide range of samples also revealed different sets of quality markers that clearly classified samples with evidence of red blood cell lysis, those with partial activation of the coagulation cascade due to inappropriate sample handling, and those with exogenous contaminations such as keratins. Even though this study provided a useful overview of the information content of the plasma proteome, the depth of coverage was not yet sufficient to address low‐level, regulatory plasma proteins. A single step of fractionation yielded a quantitative plasma proteome of about 1,000 proteins, including 183 proteins with a reported concentration of < 10 ng/ml, however at the cost of longer measurement times per sample.

An improved version of the plasma proteome profiling workflow allowed the robotic preparation and measurement of nearly 1,300 plasma proteome samples in a weight loss study (Geyer *et al*, 2016b). Quadruplicate analysis of individuals captured the dynamics of an average of 437 proteins upon losing weight and over a year of weight maintenance. Weight loss itself had a broad effect on the human plasma proteome with 93 significantly changed proteins. Quantitative differences were often small but physiologically meaningful, such as a 16% reduction of the adipocyte‐secreted factor SERPINF1. The longitudinal study design in which the individuals sustained an average 12% weight loss for 1 year allowed capturing the long‐term dynamics of the plasma proteome and categorizing it into proteins stable within versus between individuals. Multi‐protein patterns reflected the lipid homeostasis system (apolipoprotein family), low‐level inflammation, and insulin resistance. These patterns quantified the benefits of weight loss at the level of the individual, potentially opening up for individualized treatment and lifestyle recommendations.

Together, these studies also highlight the advantages of longitudinal over cross‐sectional study designs, because the plasma proteome tends to be much more constant within an individual over time than between different individuals. Furthermore, they are similar in that they use less bias‐prone undepleted plasma, and identify many proteins in a given analysis time (up to 20 proteins/min).

**Figure 5:**
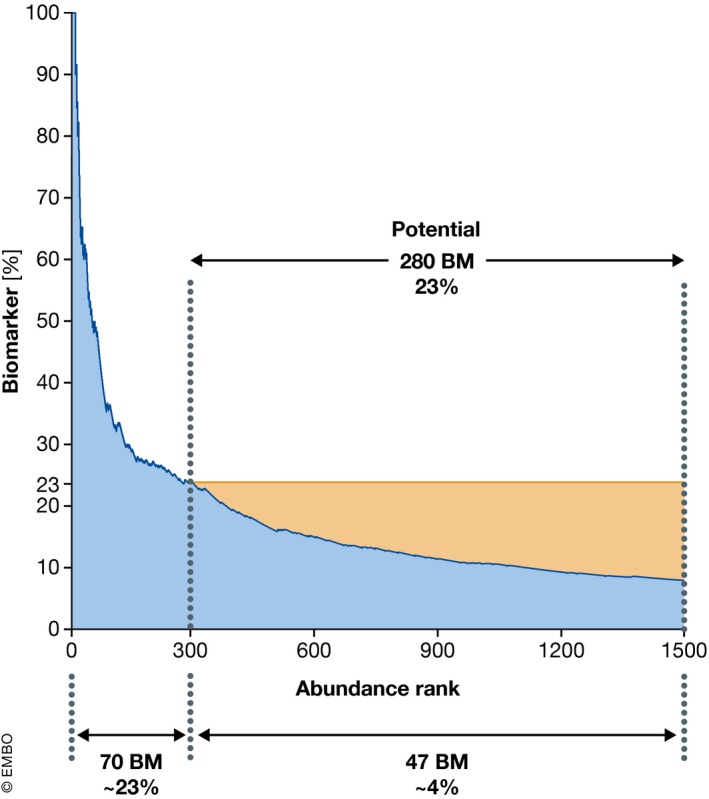
Biomarker distribution across the abundance range The blue area illustrates the percentage of biomarker (BM) as a function of increasing depth of the plasma proteome. Within the 300 most abundant proteins, 23% are already known biomarkers. The top of the yellow region extrapolates this proportion to the remainder of the plasma proteome. If the portion of biomarkers remained as high as it is in the 300 most abundant proteins, there are at least 233 potential biomarkers to be discovered (yellow area of the figure).

Regarding the question of how many proteins should be covered, we found that a proteomic depth of more than 1,500 proteins in undepleted plasma allows the coverage of tissue leakage proteins such as liver‐based lipoprotein receptors and is within reach of technological capabilities that are currently being developed. Among the first 300 highest abundant proteins, every fourth protein is a biomarker, whereas in the next 1,200 proteins, it is only every 25^th^ protein (Fig 5). As there is no *a priori* reason that biomarkers should have a skewed abundance distribution, this suggests that many biomarkers are still to be found. We believe that the real promise of plasma proteome profiling using the rectangular strategy is that it can discover proteins and protein patterns that have not been considered as biomarkers yet. The exponential increase in the underlying LC‐MS/MS technology will stimulate a matching increase in the number of plasma proteome datasets recorded in laboratories around the world. This will create an extensive database of plasma proteomes and their dynamics, involving many clinical studies and individuals. Such data could then be aggregated to build up a knowledge base that connects proteome states to a wide diversity of “perturbations”, including diseases, risks, treatments, and lifestyles. At a minimum, this approach will reveal all the different conditions in which a given set of biomarkers is involved, in addition to the specific context where they were discovered. Proteome overlap between disease conditions could reveal commonalities between them (Fig 4B, upper panel). An individual's plasma proteome profile and its dynamics could then be interpreted by comparing it to the global knowledge base. This could be used to deconvolute co‐morbidities and to guide treatment and monitor effectiveness (Fig 4B, lower panel).

## Standardization of the proteomic biomarker discovery pipeline

It has been suggested that the current lack of biomarkers making their way into the market may be the result of various technical, scientific, and political aspects including undervaluation, resulting from inconsistent regulatory standards, and lack of evidence for analytical validity and clinical utility (Hayes *et al*, 2013). To overcome these challenges, systematic pipelines for biomarker development have been advocated (Pavlou *et al*, 2013; Duffy *et al*, 2015). In the context of moving from a triangular to a rectangular strategy of biomarker discovery, it will be particularly important to consider the following principles.

(1) Analytical performance characteristics: Analytical validity is the capacity of a test to provide an accurate and reliable measurement of a biomarker. Establishment of analytical validity of the plasma proteomics methodology will be key, because the same method will often be carried on from discovery to application. Detailed standards to determine analytical validity have been developed by the Clinical and Laboratory Standards Institute (CLSI) (www.clsi.org). An overview can be found in Grant and Hoofnagle (2014) and Jennings *et al* (2009). Some of these standards have been recognized by the U.S. Food and Drug Administration (FDA) and are accepted for bringing *in vitro* diagnostic test to the market (https://www.accessdata.fda.gov/scripts/cdrh/cfdocs/cfstandards/search.cfm). Even though starting off with a full analytical validation conforming to FDA standards might be prohibitive in biomarker discovery, at least some of the key criteria, such as carryover, accuracy, precision, analytical sensitivity, analytical specificity, and limit of quantification, should be tested early on. This is in line with what we advocate in the context of the rectangular strategy and is also in the interest of saving resources, because the step following biomarker discovery is biomarker validation, where analytical validity will be mandatory.

(2) Clinical performance characteristics: Clinical validity relates to the associated diseases and clinical conditions of patients and is different from analytical validity, which focuses on the correct measurement of analytes targeted by the assay. According to International Standard Organization (ISO) 15189 and ISO 17025, validation is the “confirmation, through the provision of objective evidence, that the requirements for a specific intended use or application have been fulfilled”. Therefore, establishing clinical performance is the main goal in the validation phase of a biomarker. Clinical performance characteristics include (i) defining normal reference ranges by measuring cohorts of apparently healthy individuals, (ii) determining clinical sensitivity, which is defined as the proportion of individuals who have the disease and are tested positive, and (iii) determining clinical specificity, which is defined as the proportion of disease‐free individuals who are tested negative. Derived statistics such as receiver operating characteristic (ROC) plots are particularly helpful in assessing the clinical performance of biomarkers (Zweig & Campbell, 1993; Obuchowski *et al*, 2004).

(3) Study design and pre‐analytics: Careful study design and well‐controlled pre‐analytical conditions are key requirements at any time during a biomarker study. With respect to study design, it is mandatory to clearly define the clinical question and the medical need that should be addressed by the biomarker. A common problem in biomarker studies is that samples from cases and controls have been collected independently and are mismatched for age, ethnicity, sex, and other factors that may or may not lead to unintentional bias (Duffy *et al*, 2015). Methods against bias include proper study design as well as precise and deep clinical phenotyping of participants, using systematic classifications such as the International Statistical Classification of Diseases (http://apps.who.int/classifications/icd10/browse/2016/en) or the human phenome ontology (Kohler *et al*, 2017). In this way, if a person has multiple disease conditions, this can be properly accounted for. Sample collection is important as well, and it is imperative that all samples (including cases and controls) are treated equally from blood drawing to the analytical phase. Another critical step in many biomarker studies is biobanking. When employing ELISAs, we have found that storage of protein‐based biomarkers for 3 months requires temperatures of −80°C or below (Zander *et al*, 2014). Sample stability for longer periods is only poorly investigated. However, in our experience, shotgun proteomics has a high tolerance for variation in sample history, because there are no protein epitopes that need to be preserved and even partial protein degradation may be tolerable as long as the majority of subsequently generated proteolytic peptides remain unaltered.

## The road to clinical application

The current progress in plasma proteomics opens exciting novel avenues for research and the clinic. How likely is it, given all the aforementioned precautions that the outlined approaches will lead to the discovery of novel protein‐based biomarkers? And what will the proteomic biomarker of the future look like? A key theme in this context is the discriminative power of a biomarker to distinguish between the presence and absence of a particular disease state or risk, in other words its clinical performance. Examples of currently used biomarkers with high specificity and high sensitivity are cardiac troponins, which are structural proteins specifically expressed in cardiomyocytes and therefore highly specific for myocardial damage. For this reason, cardiac troponins have even been incorporated into the universal definition of myocardial infarction (Roffi *et al*, 2016).

It is likely that proteomics approaches will succeed in the identification of additional biomarkers with similar performance, at least for certain diseases. In fact, we need to be aware that most biomarkers used today are either highly abundant or originate from a known pathophysiological context. As a thought experiment, we have extrapolated the ratio of the number of biomarkers relative to the number of proteins in the high abundance range to lower abundance protein range, which indicates the potential for several hundred novel biomarkers, which might be accessible with appropriate technology (Fig 5). In analogy to GWAS, where a significant number of hits turned out to be related to previously unknown pathophysiology of the investigated disease (Holdt & Teupser, 2013; Manolio, 2013), it is quite likely that new markers, which have hidden below the radar of previous strategies, will be identified by novel systematic proteomics approaches. These biomarkers may also have the potential to improve our understanding of disease pathophysiology not only in diagnostics but also for therapy. Note, however, that the identified biomarkers might not always be directly involved in the disease pathophysiology but may only be associated with it.

The human genome encodes for about 20,000 protein coding genes, which is opposed to more than 14,500 diseases classified by an ICD code. This makes it even conceptually difficult to imagine that one gene or protein is associated with each disease condition, as is often implied in current efforts to find biomarkers. In contrast, the rectangular strategy, allowing to screen large cohorts for multiple markers, holds great promise to discover and validate protein patterns that are characteristic of particular health or disease states. Indeed, multi‐marker combinations may achieve higher specificity and sensitivity compared to single markers and first tools for selecting accurate marker combinations out of omics data have been developed (Mazzara *et al*, 2017). However, a common problem with new biomarkers combined with existing ones is that they frequently only lead to minor classification improvements, in particular when added to well‐performing ones (Pencina *et al*, 2010). Contrary to common and intuitive assumptions, it has been shown that correlation (especially negative correlation) between predictors can be beneficial for discrimination (Demler *et al*, 2013). More research in this area is clearly warranted, and new proteomics technologies will provide the data required for the validation of appropriate statistical methods.

Finally, how will these markers be applicable in a clinical setting? We favor in‐depth measurement of the entire plasma proteome regardless of the occasion, as this provides the most complete information. Over time, it adds to the longitudinal plasma proteome profile that could usefully be obtained even of healthy subjects. As mentioned above, plasma protein levels tend to generally be stable but person‐specific, allowing individual‐specific interpretation instead of population‐based cutoff values. Furthermore, co‐morbidities are the rule rather than the exception in many patient groups. These are much more easily and economically addressed by a generic diagnostic test such as plasma proteomic profiling rather than a succession of individual ELISA tests. Nevertheless, there would clearly be many situations in which a universal test will not be appropriate because it may inadvertently uncover other conditions. Similar issues arise with other technologies such as genome sequencing or imagining techniques, where individuals may not want to learn about predispositions that they can do little about. In these cases and generally to avoid the risk of overdiagnosis (Hofmann & Welch, 2017), clinicians may prefer plasma proteomics tests of a more directed nature that focuses on a particular disease context. This could be accomplished by the above‐mentioned MS techniques targeting a panel of proteins, rather than the entire proteome.

**Figure 6:**
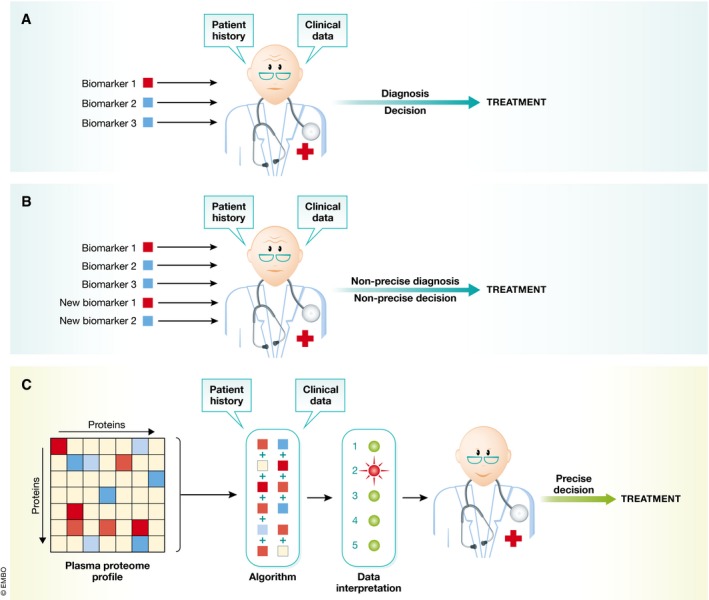
Implementation of proteomic data in clinical decisions (A) Currently, physicians make treatment decisions on the basis of a few plasma biomarker tests, combined with patient history and clinical data (upper panel). (B) Adding new biomarkers would quickly overwhelm the current paradigm—leading to suboptimal clinical decisions. (C) Multi‐protein panels and the data from past studies (the knowledge base in Fig 4B) are combined algorithmically. This will aid the physician in making more precise recommendations for treatment, while still taking patient history and other clinical data into account.

For either whole‐proteome diagnostic tests or panel‐based tests, the question arises how doctors would deal with the resulting multi‐dimensional data. Figure 6A shows the current single/oligo biomarker diagnostics, which is integrated into decision‐making largely based on clinical knowledge and intuition. New biomarkers clearly hold the promise of better informed clinical decisions, but also imply the risk of generating patterns exceeding the human cognitive capacity of interpretation (Fig 6B). A solution to this problem might be the algorithmic combination of multiple biomarkers into a quantitative panel, possibly combined with clinical metadata, which might substantially aid clinical decision‐making (Fig 6C). Given rapid developments in “deep learning” and “big data”, it will be very interesting to see whether this combination can provide powerful and unprecedented associations. We note that there are already multi‐parameter scores in clinical practice today. For instance, the Child–Pugh score and the Framingham Risk Score have each combined several blood values with patient data, to aid clinician's decision in treating liver disease and cardiovascular treatment, respectively, for decades. This also suggests a way how plasma proteomics could be accepted into evidence‐based medical practice, a huge challenge given the many parameters and parameter combinations involved, which clearly cannot all be validated with separate clinical trials. A pragmatic alternative might be to devise trials in which doctors randomly obtain the proteomic information and associated decision support. It would then be straightforward to determine whether there is a significant benefit in patient outcomes.

## Conclusions

Staking stock of the current practice in laboratory medicine shows that the majority of treatment decisions are made on the basis of blood tests and that protein measurements are even today the most prominent among them. Despite successfully being carried out by the millions every year, these assays are almost always directed against individual proteins and the pace of introduction of new protein tests has slowed to a trickle.

MS‐based proteomics clearly has the potential for multiplexed and highly specific measurements, in which protein patterns rather than single biomarkers could be the relevant readout. Our review of the literature revealed that past efforts were held back by the great analytical challenges of the plasma proteome, something that is only now giving way to exciting technological developments. We argue that the analysis of large numbers of conditions and participants in all stages of the discovery and validation process has the potential to produce biomarker panels that are likely to be of clinical value. When coupled to large knowledge bases of changes in protein patterns in defined conditions, such a plasma proteome profiling strategy could in principle exploit the entire information contents of this body fluid.

To make this vision a reality, further improvements in throughput, depth of proteome coverage, robustness, and accessibility of the underlying workflow are crucial. Furthermore, plasma proteomics can also be extended to the analysis of post‐translation modifications. Likewise, plasma metabolomics also uses MS‐based workflows and could routinely be integrated with plasma proteomics in the future. We are confident that the required technological developments can and will all be achieved over time. At least as much of a challenge will be conceptual and “political”, as the proteomic information deluge needs to be turned into actionable data for the physician and the healthcare system. This will require a dedicated and untiring commitment from all partners involved. We believe that the promise of much more precise and specific diagnostics will amply reward such efforts.

## Conflict of interest

The authors declare that they have no conflict of interest.

## Supporting information



Dataset EV1
